# Effects of amines on the formation and photodegradation of DCNM under UV/chlorine disinfection

**DOI:** 10.1038/s41598-020-69426-9

**Published:** 2020-07-28

**Authors:** Lin Deng, Xueying Liao, Jiaxin Shen, Bohui Xu

**Affiliations:** Department of Municipal Engineering, Southeast University, Nanjing, 210096 People’s Republic of China

**Keywords:** Environmental sciences, Environmental chemistry, Environmental impact

## Abstract

Investigations were conducted to examine the effects of amine type and initial concentration, free chlorine concentration, UV light intensity, pH and *tert*-butyl alcohol (TBA) on the formation of dichloronitromethane (DCNM) under UV/chlorine. Methylamine (MA), dimethylamine (DMA) and poly-dimethyl diallyl ammonium chloride (PolyDADMAC) were selected as the amine precursors of DCNM. And the reaction products of amines were explored through observing the contents of various nitrogen under UV/chlorine. Experimental results indicated that the higher of the intensity of UV light, the concentration of amines and free chlorine, the greater of the amount of DCNM formation; the amine substance with simple structure is more likely oxidized to form DCNM, so the potential of MA to form DCNM is the largest among three amines; the formation of DCNM decreased with increasing pH from 6.0 to 8.0; due to adding TBA into the reaction solution, halogen and hydroxyl radicals were restrained which resulted the DCNM formation decreased. In the reaction process, the formation of DCNM from amines increased at the beginning, then decreased and almost disappeared due to photodegradation. During the formation and photodegradation of DCNM, the dissolved organic nitrogen could be transformed into the ammonia-nitrogen (NH_3_-N) and nitrate-nitrogen (NO_3_^−^-N).

## Introduction

In the past 10 years, more and more attention has been paid to the safety of drinking water supply. Disinfection of public water sources is one of the important ways to protect people's health. It is significant to reduce the occurrence and spread of waterborne diseases in water^1^. And the impacts of many water-related infectious diseases have been greatly reduced through disinfection of drinking water^2^. Therefore, urban water supply system can provide quality drinking water to residents every day, and meet people's daily needs. However, chemical disinfection also poses a severe issue to us that forming new chemical substances which called disinfection by-products (DBPs). Chlorine is also commonly used in the water treatment process, which can maintain a certain residual chlorine of the effluent to keep the subsequent disinfection. In addition, chlorine also can inactivate waterborne pathogens so that it can reduce the public health risk^3^. But in the 1970s, previous research confirmed that chloroform would form during the chlorine disinfection process, which means that chlorination would bring a range of toxic DBPs^4,5^. Since then, DBPs attracted extensive attention from all walks of life. To date, more than 800 DBPs have been reported, and most of these were identified in the laboratory by simulated disinfection studies^6^. Besides, several epidemiological studies have shown that prolonged exposure to some DBPs will pose a risk of carcinogenicity. Some of these may lead to bladder cancer or colorectal cancer^7^. Even there are some potential health problems from DBPs for humans, such as developmental and reproductive complications^8^.

About DBPs, a lot of studies have been searched about carbonaceous DBPs (C-DBPs), especially trihalomethanes (THMs) and haloacetic acids (HAAs). Compared with THMs and HAAs, the content of nitrogenous DBPs (N-DBPs) in water always at a lower level, resulted in less research about N-DBPs in the initial study on DBPs^4,9^. For instance, in drinking water distribution systems (DWDSs), halonitromethanes (HNMs) level is in between 0.16 and 1.50 mg/L and generally higher in summer than in winter or spring^10,11^. In swimming pools, HNMs is in the range of 0.2 to 0.7 μg/L^12^. N-DBPs have lately received great attention for their higher genotoxicity and cytotoxicity than those of regulated C-DBPs^13,14^. Among N-DBPs, HNMs, haloacetonitriles (HANs), haloacetamides (HAcAms), and N-nitrosamines (NAs) are frequently detected in drinking water and wastewater, which brings serious health risks to human^9^. Toxicological research has uncovered that the cytotoxicity and genotoxicity of HAN and HNM are 1–2 orders of magnitude higher than their haloacetic acid analogs^15^. Zhang’s group found that HNMs can also cause oxidative damage of DNA in mice^16^. Thus, many regions have begun monitoring HNMs in drinking water to protect human health. As one typical class of HNMs, DCNM has the characteristics of low concentration levels (average 1.0 mg/L in DWDSs), low reported frequency and high toxicity (cytotoxicity value: 3.73 × 10^–4^; genotoxicity value: 4.21 × 10^–4^)^17,18^. At present, the precursors and the formation mechanism of HNMs are still at the stage of exploration. Bond et al. proposed that there was still great uncertainty about the characteristics of chemical group of HNMs precursors in drinking water (hydrophobicity or hydrophilic), and the key factor might be whether there were functional groups which can convert to nitro (e.g. amines or phenols)^19^. This study also claimed that dissolved organic nitrogen (DON) was the most relevant parameters to the formation of HNMs. And it has been reported that nitrite was potential source of nitrogen for the formation of chloropicrin^20^. Liew et al. research confirmed the increase of HANs formation in water with high content of NH_3_-N due to monochloramine formation from the chlorination of ammonia^17^. There is an important link between NH_3_-N and the formation of HANs.

UV disinfection is a commonly used disinfection method in water treatment, which can effectively inactivate various microorganisms in water. It has the advantages of fast sterilization, no harmful DBPs, no need for additional chemical drugs, and it does not introduce disinfectant resistance to bacteria^21^. However, in actual operation, there are some problems of UV disinfection such as low disinfection efficiency, no subsequent anti-virus ability, and unstable disinfection test results. So it is usually coupled with chlorine in water treatment^12,22,23^. As an emerging advanced oxidation process (AOP), UV/chlorine treatment can produce diverse reactive species which are beneficial to degrade a variety of contaminants^24^. Most studies believed that UV treatment can reduce the concentration of DBPs in drinking water^25^. But other studies showed that the formation of N-DBPs was related to the chlorination of natural organic matter (NOM), and UV treatment can degrade NOM into lower molecular weight products, which could react with chlorine or chloramine to promote the formation of DBPs^5,26–29^. For example, during the UV–chlorination process, THMs formed were higher than chlorine alone treatment, and the formation of HANs and HNMs also increased^30,31^. Lu’s group specified UV/chlorine pre-treatment of clofibric acid (CA) could promote the concentration of TCNM^32^. And in previous research, we did found that TCNM formation increased by UV–chloramination compared by chlorination alone^33^. It also has been reported UV–chloramination process formed the DBPs which are more cytotoxic than those by chloramination alone^34^.

Amines are widely distributed in water environments due to decomposition of protein in domestic sewage and extensive use of various amines as chemical raw materials^35^. During the treatment of sewage or drinking water, primary and secondary amines can react with disinfectants to form carcinogenic and mutagenic nitrosamines, which brings potential health risk. Until now, there are few reports on the effect of amines in water on the formation of DCNM under UV/chlorine disinfection. Therefore, in this study, three different classes of amines (MA, DMA and PolyDADMAC) were selected as the precursor of DCNM. The effects of amines concentration, free chlorine concentration, light intensity, pH and additional TBA on DCNM formation were explored with three different amine precursors. The DCNM formation potential of three amine precursors and the variation of different forms of nitrogen in the reaction system was analysed.

The aims of the present study were (1) to evaluate the effects of amine on the formation and photodegradation of DCNM under UV/chlorine with influencing factors of light intensity, free chlorine, pH and TBA and reaction time, etc., (2) to analyse the formation and photodegradation of DCNM from amine under UV/chorine, (3) to explore DON could be transformed into ammonia-nitrogen (NH_3_-N) and nitrate-nitrogen (NO_3_^−^-N) during the formation and photodegradation of DCNM, (4) to compare the formation and photodegradation of DCNM in three different amine precursors. In order to better show the phenomena of these experiments and the requirements of instrument detection, the concentrations of chlorine and amine used in these experiments were slightly larger than that in the actual water body. The results of the current study could be very useful for controlling HNMs formation and contributing to the development of new disinfection method in the drinking water and sewage treatment.

## Materials and methods

### Chemicals and reagents

Sources of chemicals and reagents were provided in the Supplementary Text S1.

### Experimental equipment

**Figure 1:**
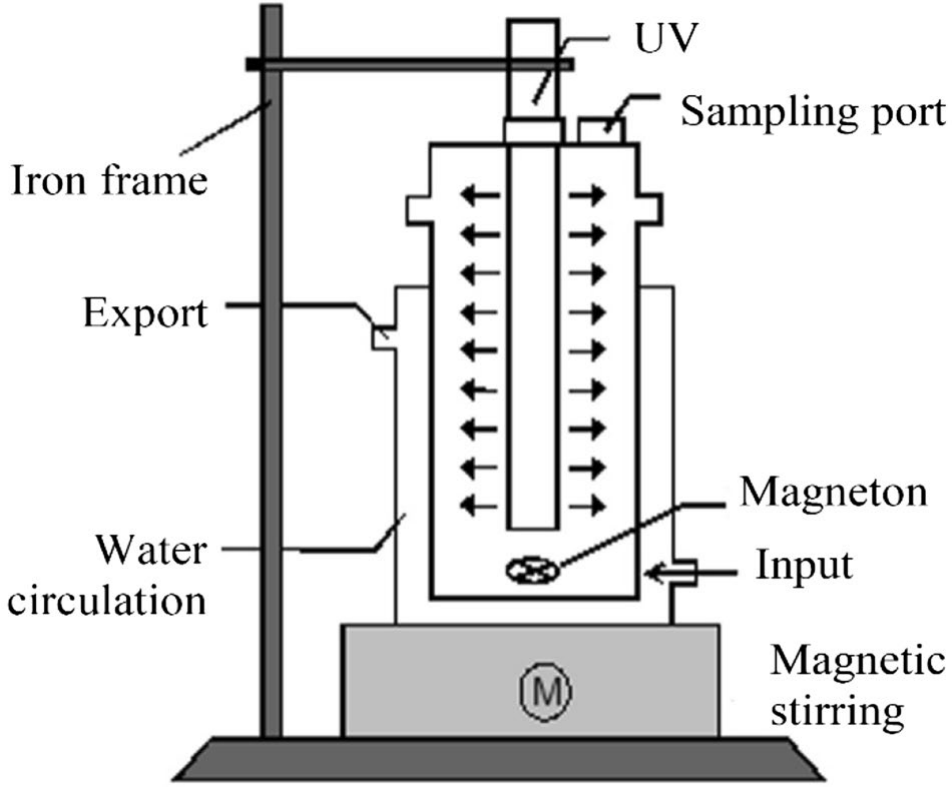
Schematic diagram of the reactor.

The experiments were carried out in a self-made quartz glass reaction equipment which has two layers (see Fig. 1). The outer layer was used for the condensing water circulation to maintain the constant temperature of reaction solution (22 ± 2 °C), and the reaction proceeded in the inner layer with the conventional low-pressure UV mercury lamps (5, 10, 15 W), which emits almost monochromatic light at 254 nm. A magnetic stirrer was arranged under the reactor to ensure a uniform mixture of the reaction solution. Before conducting the experiment, the equipment should be placed in a box so that the reaction could be carry out under a dark condition.

### Experimental methods and analyses

According to the experimental requirements, the initial reaction solution with different concentration was prepared for adding into the reaction device. The UV lamp was immersed in the reaction solution, while the height of the tube holder was adjusted to control the lamp to a suitable depth. After the reaction started, 5.0 mL of the reaction solution was transferred to a brown sample vial with cap, at regular intervals. 2 mL methyl tert-butyl ether (MTBE) was used to liquid–liquid extraction (LLE) before analysis. Then, taking 1 mL MTBE to a gas chromatography (GC) vial from the extracted solution and then analysed the samples using GC-ECD system. The initial operating temperature of GC was 50 °C. After the instrument was operated at the initial temperature for 5 min, the temperature was raised to 140 °C at 10 °C/min, and then raised to 280 °C at 20 °C/min. Among them, the temperature of the inlet of instrument was 235 °C and the ECD was 280 °C. Nitrogen was the carrier gas of GC, and the flow rate was 1.0 mL/min. In addition, in this experiment, alkaline potassium persulfate digestion UV-spectrophotometry method was used for determination of total nitrogen (TN); NH_3_-N was determined by Nessler’s reagent spectrophotometry; NO_3_^−^-N and nitrite-nitrogen (NO_2_^−^-N) were determined by ultraviolet spectrophotometry. All experiments were performed at least twice, and error bars represent one standard deviation of the average values.

## Results and discussion

### Effects of amine type and initial concentration

**Figure 2:**
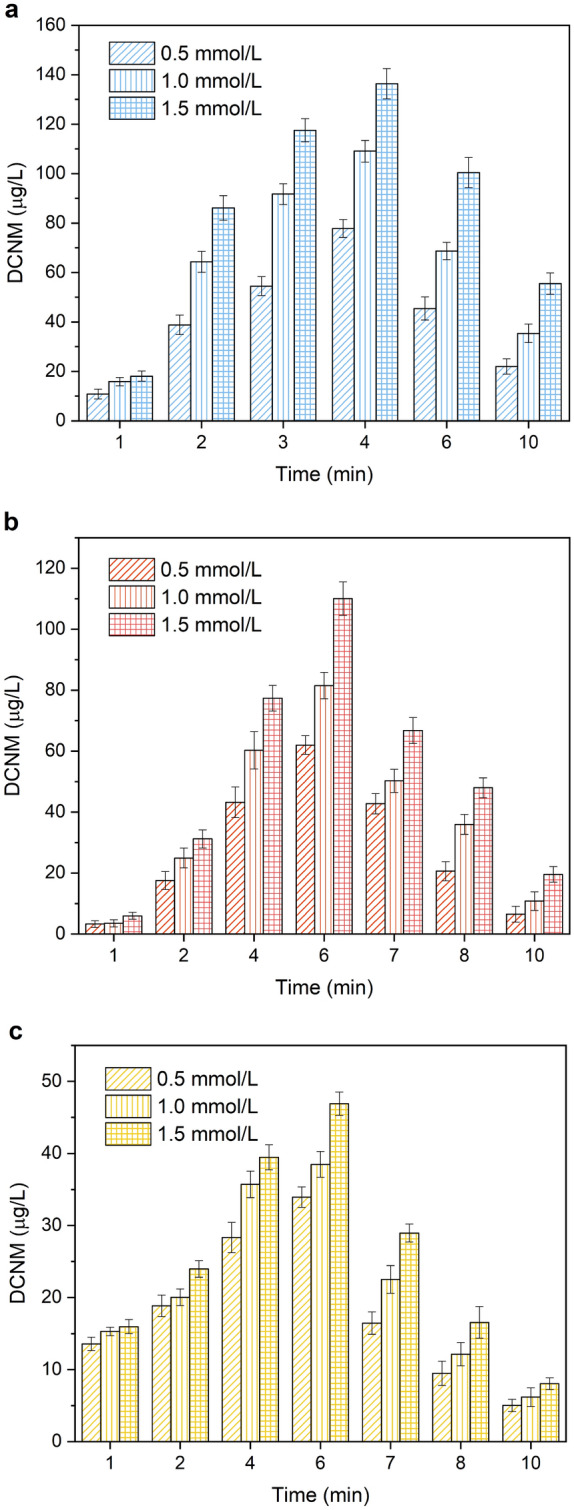
Effect of the initial concentration of (**a**) MA, (**b**) DMA and (**c**) PolyDADMAC on the formation and photodegradation of DCNM under UV/chlorine.

In order to investigate the DCNM formation potential of amine precursors, the solutions of MA, DMA and PolyDADMAC containing the organic nitrogen of 0.5, 1.0 and 1.5 mmol/L were prepared for the experiments, respectively. And the reaction solution was subjected to 15 W UV irradiation under the condition of 60 mg/L free chlorine at pH 7. From Fig. 2a, when MA acted as precursor of DCNM, the concentration of DCNM formation increased quickly at the beginning, and reached the maximum at 4 min, then the declined tendency of DCNM formation was observed after that. As shown in Fig. 2b,c, when DMA and PolyDADMAC acted as precursor of DCNM, DCNM formed reached the maximum at 6 min, and then decreased with the increase of reaction time. When the reaction solution contained 0.5 mmol/L organic nitrogen, in the presence of MA, DMA, PolyDADMAC, the maximum amount of DCNM formation was 77.83, 61.98 and 33.92 μg/L respectively, and then decreased to 21.95, 6.50 and 5.02 μg/L at 10 min. When the organic nitrogen concentration of MA, DMA, PolyDADMAC was 1.0 mmol/L, the maximum production of DCNM was 109.10, 81.48 and 38.48 μg/L, respectively. When the organic nitrogen concentration of MA, DMA, PolyDADMAC increased to 1.5 mmol/L, the maximum production of DCNM was 136.36, 110.02 and 46.90 μg/L. Compared to the reaction with 1.0 mmol/L organic nitrogen, the maximum amount of DCNM formed increased 25.0%, 35.0% and 21.9% respectively. Thus it was demonstrated that the increase of precursor concentration can promote the formation of DCNM. At the same time, the formation of DCNM from simple structure amines was higher. During UV/chlorine process, UV irradiation can provide energy for the bond-breaking and ring cleavage of PolyDADMAC to destroy its structure^36,37^. Therefore, PolyDADMAC would be degraded into simple structure amines firstly, and then continue to react with free chlorine to form DCNM. Polymers such as PolyDADMAC required more energy and oxidants to degrade into low-molecular products than amines with simple structure, which caused the concentration of DCNM formation by the three amines is MA > DMA > PolyMAMDAC, under the same experimental conditions.

### Effect of free chlorine concentration

**Figure 3:**
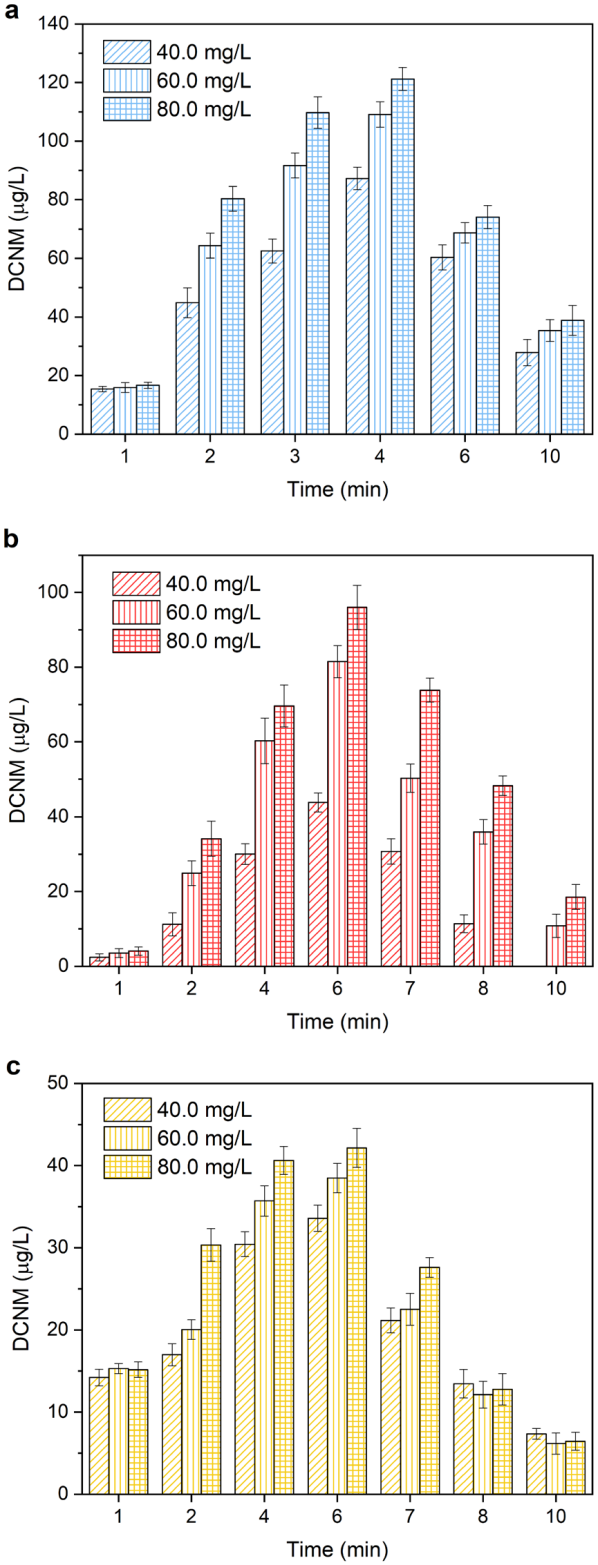
Effect of free chlorine concentration on the formation and photodegradation of DCNM from (**a**) MA, (**b**) DMA and (**c**) PolyDADMAC under UV/chlorine.

Free chlorine plays a vital role in the process of DCNM formation under UV/chlorine. The reaction solutions containing the organic nitrogen of 1.0 mmol/L (MA, DMA and PolyDADMAC, respectively) were prepared for the experiments, respectively. The effect of free chlorine concentration on forming DCNM was investigated by adding 40.0, 60.0 and 80.0 mg/L free chlorine into the reaction solutions, respectively. As shown in Fig. 3, when the concentration of free chlorine was 40 mg/L, the maximum amount of DCNM formation was 87.28, 43.78 and 33.59 μg/L in the reaction solutions of MA, DMA and PolyDADMAC, respectively. When the concentration of free chlorine was 60.0 mg/L, the maximum production of DCNM was 109.1, 81.48 and 38.48 μg/L from MA, DMA, PolyDADMAC respectively, which increased 25.0%, 86.1%, 14.6% in comparison with 40 mg/L free chlorine. When the concentration of free chlorine reached 80.0 mg/L, the amount of DCNM formation continued to up to 121.24, 96.02 and 42.16 μg/L respectively, which increased 38.0%, 119.3%, 25% in comparison with 40 mg/L free chlorine. It also can be found that the formation of DCNM from DMA was the most sensitive to the change of chlorine dose, followed by MA, PolyDADMAC. Therefore, the experiment results showed that the concentration of DCNM formation was positively correlated to the concentration of free chlorine in the presence of amine. It was different from the result of chloramphenicol (CAP) as a precursor which indicated the formation of DCNM from CAP decreased with the increase of chlorine dose. As chlorine increased, the rate of degradation of CAP would decrease, resulting in the decrease of free radicals produced by CAP degradation^31^. However, for amines, the high concentration of free chlorine could accelerate the breaking of bond and oxidation process, which might contribute to subsequent chlorination of the intermediates. So, the concentration of DCNM formed by amines increased with the increase of chlorine.

### Effect of UV light intensity

**Figure 4:**
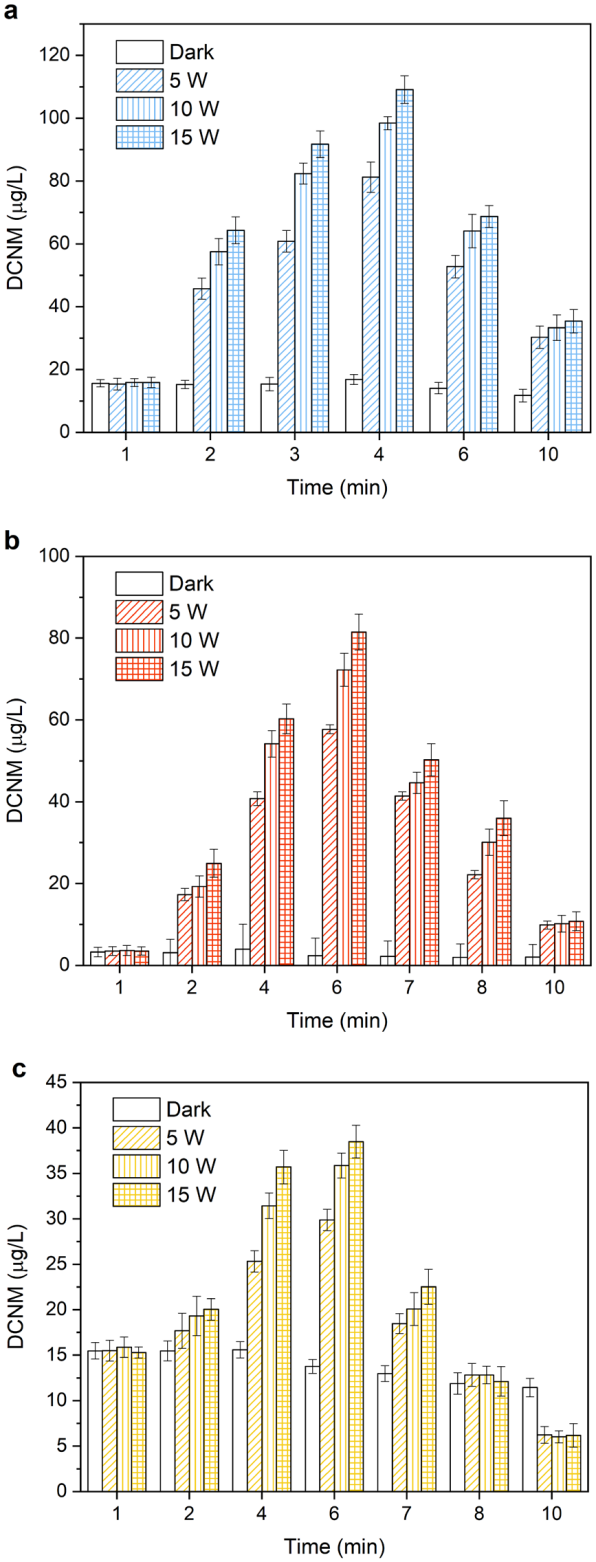
Effect of UV light intensity on the formation and photodegradation of DCNM from (**a**) MA, (**b**) DMA and (**c**) PolyDADMAC under UV/chlorine.

Low pressure UV could degrade amines into lower molecular weight products, which react with chlorine to promote the formation of DBPs. And the photolysis of free chlorine using conventional UV mercury lamps could produce reactive oxygen species (e.g., ozone and HO·) and reactive chlorine species (RCS), which will promote DBPs formation^38–40^. To investigate the influence of UV light intensity on the formation and photodegradation of DCNM from MA, DMA and PolyDADMAC, the reaction solution (MA, DMA and PolyDADMAC) containing 1.0 mmol/L and free chlorine 60 mg/L at pH 7.0 and 22 °C under 5, 10, and 15 W irradiation, respectively. In addition, each precursor group was set up with a control experiment under dark condition. As shown in Fig. 4, the amount of DCNM formation from DMA fluctuated between 1.98 and 3.95 μg/L under dark condition, while the amount of DCNM formation from MA and PolyDADMAC was about 11.71–16.81 μg/L and 11.44–15.59 μg/L, respectively. When MA, DMA and PolyDADMAC were used as precursors of DCNM, the formation of DCNM increased rapidly under UV irradiation, and reached the maximum at 4 min, 6 min, 6 min respectively. Under 5 W UV irradiation, in the presence of MA, DMA and PolyDADMAC in the reaction solution respectively, the maximum concentration of DCNM was 81.20, 57.69 and 29.87 μg/L. The maximum concentration of DCNM was 98.42, 72.23, 35.85 μg/L respectively under 10 W UV irradiation. Under 15 W UV irradiation, the maximum formation of DCNM from MA, DMA and PolyDADMAC was 109.10, 81.48 and 38.48 μg/L respectively. In comparison with 10 W UV irradiation, DCNM formation increased 10.9%, 12.8% and 7.3% under 15 W UV light intensity. According to the experimental result, DMA was the most affected by the UV light intensity among three types of amine precursors. When light intensity increased from 5 to 15 W, the increase percentage of DCNM formation from DMA was the largest (41.2%), followed by MA (34.4%), PolyDADMAC (28.8%). These phenomena were similar with the results reported by Wang's group, which described the formation of TCNM increased with the increase of UV irradiation intensity^41^. UV light irradiation played an important role in the formation of DCNM, in addition, the higher the light intensity of UV was, the faster the formation and photodegradation of DCNM was. The reason might be the increase of light intensity promoted the oxidation of amines and was helpful for DCNM formation, while the photodegradation of DCNM increased with the increase of light intensity because of its nitrile (–CN) and nitro (–NO_2_) functional groups with appropriate optical absorption properties.

**Figure 5:**
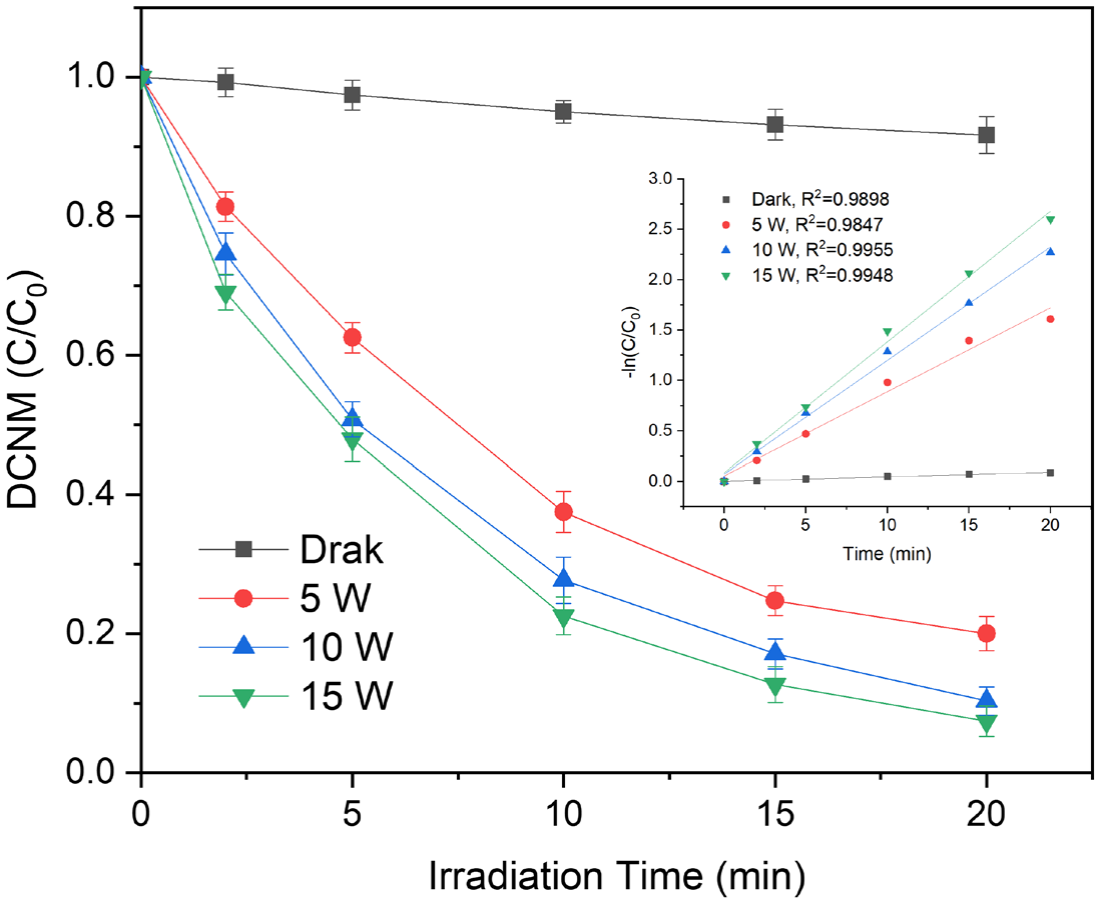
Effect of light intensity on the photodegradation of DCNM.

To explore the reasons why the concentration of DCNM formation decreased gradually after the maximum concentration in Figs. 2, 3 and 4, the effect of low-pressure UV irradiation on the photodegradation of DCNM was investigated. The experiments were carried out in 100 µg/L DCNM solution at pH = 7.0 under different light intensity (5, 10 and 15 W). The results showed that the photodegradation efficiency of DCNM increased with increasing light intensity. As shown in Fig. 5, the degradation percentage of DCNM was 8.33% after 20 min without UV. It demonstrated that DCNM in solution could be volatilized or hydrolyzed under dark condition. After 20 min, the degradation percentage of DCNM by 5 W UV irradiation was 79.98%, while that by 10 W and 15 W UV irradiation was 89.67% and 92.57%, respectively. The DCNM degradation data of all four conditions were processed by the pseudo-first-order kinetics model (Eq. (1)), and the insert figure in Fig. 5 shows that four linear fitting curves (with R^2^ > 0.98) were obtained. The *k*_obs,T_ values of UV treatment (5, 10, 15 W) was 0.0833, 0.1127, 0.1297 min^−1^ respectively, which was 19–29 folds that observed from the dark condition (0.0045 min^−1^). The *k*_obs,T_ was related to UV light intensity and increased with the increase of light intensity. It can found that UV can effectively promote the DCNM degradation and high UV light intensity providing more energy to promote photodegradation of DCNM.

1 $$- \frac{{\ln \left[ {DCNM} \right]_{t} }}{{\ln \left[ {DCNM} \right]_{0}}} = k_{obs,T} t$$

where *k*_obs,T_ is the pseudo-first-order rate constants (min^−1^).

### Effect of pH

**Figure 6:**
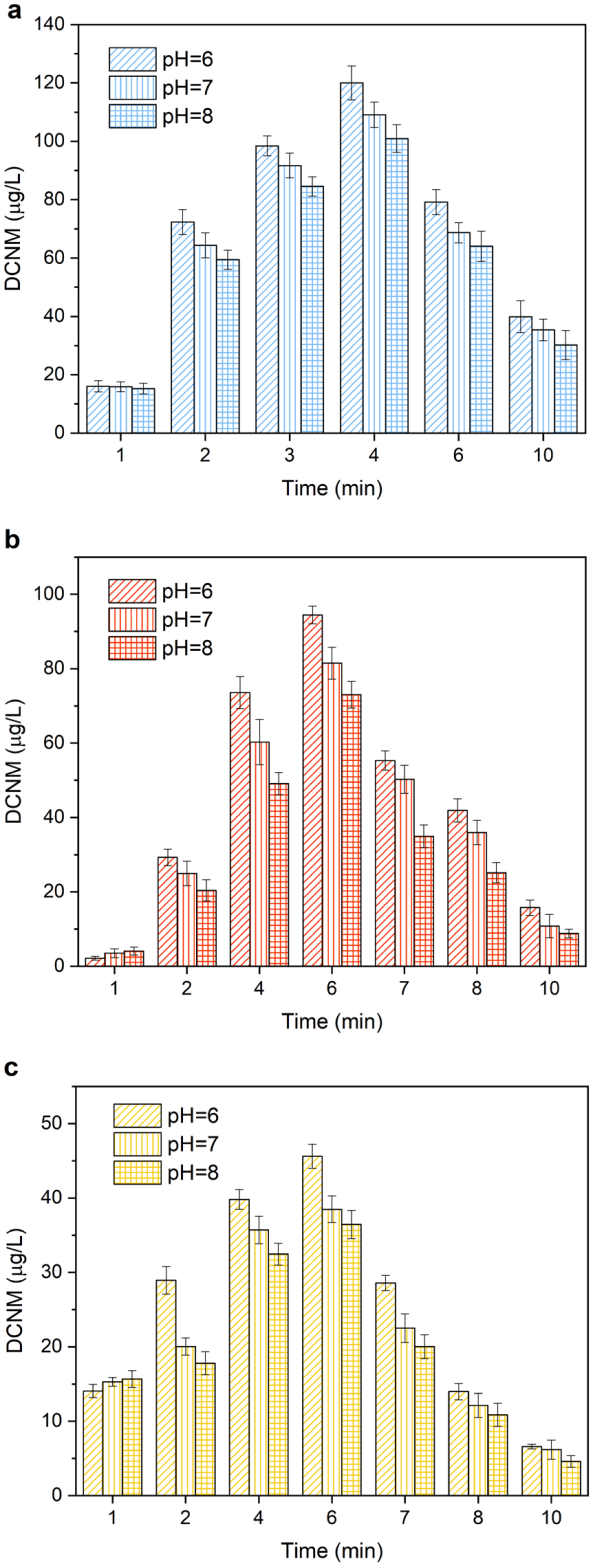
Effect of pH on the formation and photodegradation of DCNM from (**a**) MA, (**b**) DMA and (**c**) PolyDADMAC under UV/chlorine.

The pH of natural water or water plant effluents generally ranged from 6.0 to 8.0. To study the formation and photodegradation of DCNM at different pH values, the experiments were carried out in reactive solutions containing 60 mg/L free chlorine and 1.0 mmol/L organic nitrogen (MA, DMA and PolyDADMAC, respectively) under 15 W UV irradiation at pH 6.0, 7.0, and 8.0. From Fig. 6a, at pH 6.0, the maximum concentration of DCNM formation reached 120.04 μg/L in the presence of MA. When DMA and PolyDADMAC worked as the precursors, the maximum concentration of DCNM was 94.43 and 45.62 μg/L respectively (from Fig. 6b,c). At pH 7.0, the maximum concentration of DCNM formation from MA, DMA or PolyDADMAC decreased to 109.10 81.48 and 38.48 μg/L respectively. At pH 8.0, the maximum formation of DCNM from MA, DMA or PolyDADMAC decreased to 100.97, 73.01 and 36.45 μg/L respectively, which decreased 15.9%, 22.7%, 20.1% compared to the condition of pH 6.0. And DMA was the most affected by pH among three types of amine precursors. The experimental results revealed that the formation of DCNM decreased with increasing pH from 6.0 to 8.0. This is similar to the research results of Dong et al. that the acidic condition was more favorable for the generation of chloronitromethane (CNM) during UV/chlorine disinfection process^31^. The concentration of hydroxyl radical (HO·) and chlorine free radicals (Cl·) showed a decreased trend with the increase of pH, which might be the reason of the decrease of DCNM formation^42^.

### Effect of TBA

**Figure 7:**
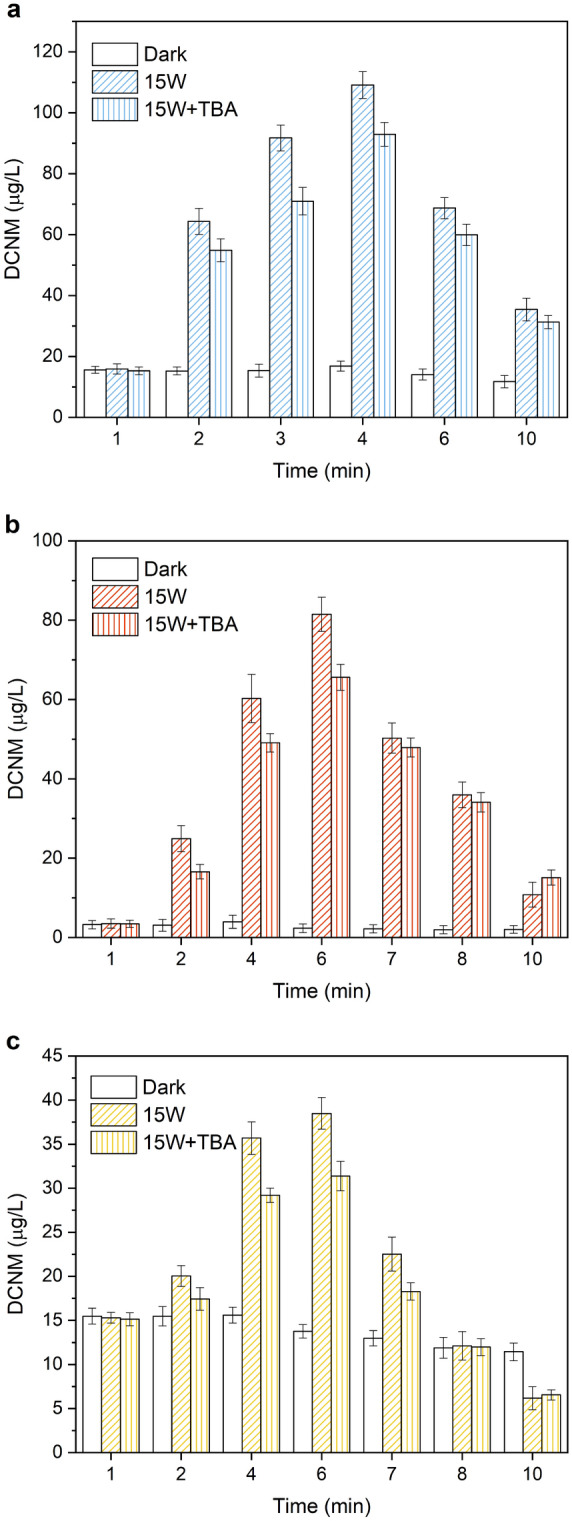
Effect of TBA on the formation and photodegradation of DCNM from (**a**) MA, (**b**) DMA and (**c**) PolyDADMAC under UV/chlorine.

TBA was selected as free radical quenching reagent to restrain HO**·** and Cl· under UV/chlorine. The experiments were conducted in the reaction solutions which contained 1.0 mmol/L organic nitrogen (MA, DMA and PolyDADMAC, respectively) and free chlorine (60 mg/L) at pH = 7, and 15 W UV irradiation. To ensure halogen and hydroxyl radicals completely suppressed in the reaction process, 5.0 g/L TBA was added in the reaction solutions. The experimental results were shown in Fig. 7, it illustrated that TBA had an important effect on the formation and photodegradation of DCNM. Under dark condition, the maximum concentration of DCNM formation from MA, DMA and PolyDADMAC was to 16.81, 3.95 and 15.59 μg/L, respectively. And with 15 W UV irradiation, in the reaction solution without TBA, the maximum formation of DCNM was to 109.1, 81.48 and 38.48 μg/L respectively. With 15 W UV irradiation and adding excess TBA, the maximum concentration of DCNM formation from MA, DMA and PolyDADMAC was 92.92, 65.59 and 31.37 μg/L, which decreased 14.8%, 19.5% and 18.5% compared to the absence of TBA. In the reaction process, the oxidation processes of precursors were hindered due to halogen and hydroxyl radicals being restrained, which resulted in the decrease of DCNM formation. Due to photodegradation, the concentration of DCNM dropped to 31.27, 15.10 and 6.55 μg/L at 10 min, respectively. However, compared to the absence of TBA, the final concentration of DCNM from DMA and PolyDADMAC was larger. This might be due to the presence of radicals scavenging compounds (TBA), which led to the weakening of the contribution of hydroxyl radicals to DCNM degradation. Therefore, it can be concluded that TBA could change the distribution of halogen and hydroxyl radicals in the solution might change, then affecting the formation and photodegradation of DCNM from amines.

### Changes of various forms of nitrogen from MA, DMA and PolyDADMAC under UV/chlorine

**Figure 8:**
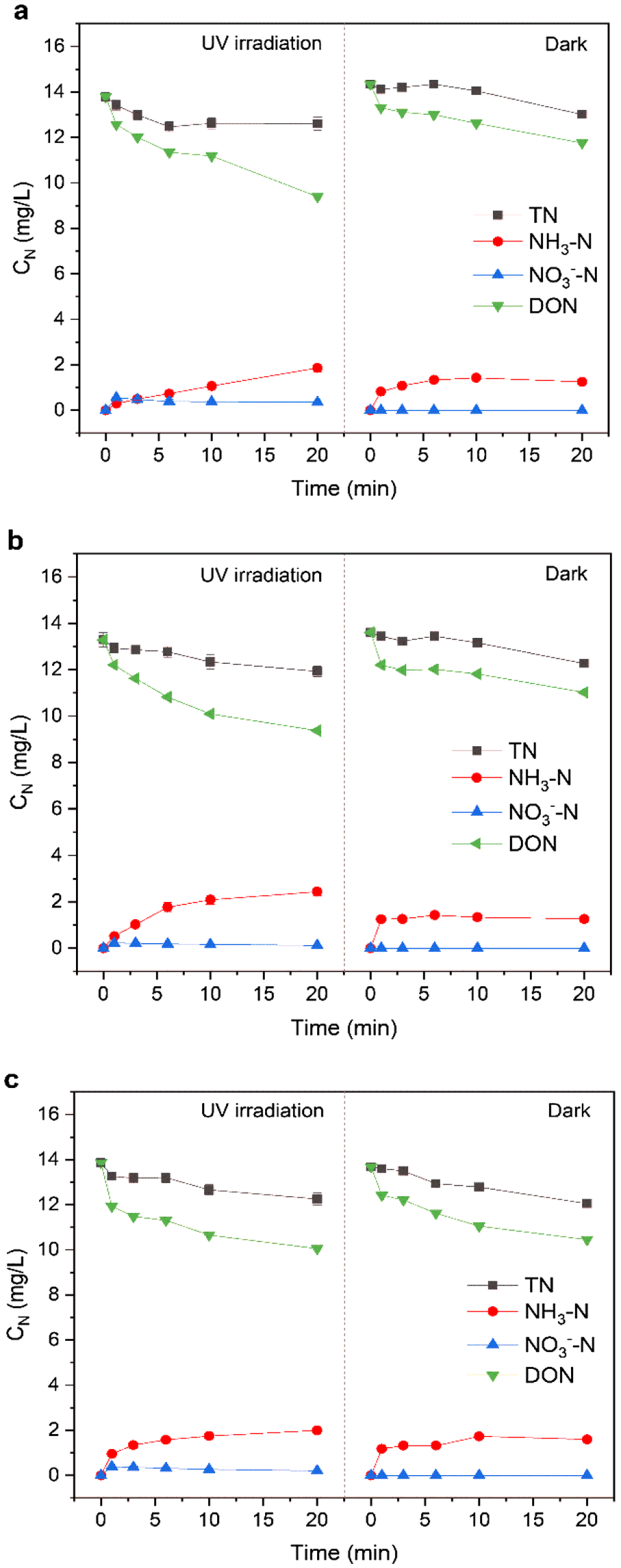
Changes of various forms of nitrogen from MA, DMA and PolyDADMAC under UV irradiation and dark condition ((**a**) MA, (**b**) DMA, and (**c**) PolyDADMAC).

To study the final products of the reaction further, a correlation between the formation and photodegradation of DCNM and the various forms of nitrogen under UV/chlorine was investigated. The experiments were carried out in the reaction solution containing 1.0 mmol/L organic nitrogen (MA, DMA and PolyDADMAC, respectively) and 60.0 mg/L free chlorine at pH 7.0 under 15 W UV irradiation and dark condition. As shown in Fig. 8, it showed that the TN had a declining trend with the increase of reaction time. With 15 W UV irradiation, the concentration of TN from MA, DMA and PolyDADMAC decreased from 13.78, 13.29 and 13.87 to 12.61, 11.93 and 12.26 mg/L respectively, and decreased by 1.17, 1.36, 1.61 mg/L in 20 min. And under dark condition, the concentration of TN also decreased by 1.33–1.63 mg/L. The loss of TN might be due to the formation of volatile nitrogen-containing organic compounds and N_2_ in the reaction process. At the beginning, NH_3_-N did not present in the reaction solution. And then, at 20 min, the concentration of NH_3_-N from MA, DMA and PolyDADMAC increased to 1.86, 2.44, 2.00 mg/L respectively, which was slightly higher than that in dark conditions (1.25, 1.26, 1.60 mg/L). The NO_3_^−^-N concentration from MA, DMA and PolyDADMAC increased initially from 0 to 0.56, 0.22 and 0.38 mg/L in 2 min and then decreased to 0.36, 0.12 and 0.20 mg/L at 20 min respectively. However, NO_3_^−^-N was not detected under dark condition. Due to the condition of free chlorine, the NO_2_^−^-N in the reaction solution was considered to be at a negligible level. Therefore, DON in the reaction solution was calculated via Eq. (2).

2 $$C_{DON} = C_{TN} - C_{{NH_{3} - N}} - C_{{NO_{3}^{ - } }}$$

According to calculated results, DON in the reaction solution had a large decrease during the process of UV/chlorine disinfection. With 15 W UV irradiation, the concentration of DON from MA, DMA and PolyDADMAC decreased from 13.78, 13.29, 13.87 to 9.40, 9.37, 10.06 mg/L respectively, decreased by 4.39, 3.92, 3.81 mg/L in 20 min, which showed that amines and DON could be decomposed into inorganic matter by oxidizing reaction. Under dark condition, DON from MA, DMA and PolyDADMAC also showed a decreased trend, which declined 2.58, 2.59, 3.23 mg/L in 20 min. Through comparison of DON between UV irradiation and dark condition, it was found that more DON participated in the UV/chlorine process. From the results, it can be seen that the DON in the reaction solution would eventually be converted into NH_3_-N and NO_3_^−^-N during the UV/chlorine disinfection process. And NO_3_^−^-N would react further with other dissolved organic matter or produce reactive nitrogen species (RNS) by UV photolysis, which led the concentration of NO_3_^−^-N in the reaction solution to rise firstly and then fall.

## Conclusions

The experimental results showed that MA, DMA and PolyDADMAC were important precursors for DCNM formation. The combined UV/chlorine condition could promote the formation and photodegradation of DCNM from MA, DMA and PolyDADMAC in contrast to free chlorination alone. The increase of amine concentration, free chlorine concentration and UV light intensity could effectively increase the formation of DCNM. The production of DCNM decreased with increasing pH value. The halogen and hydroxyl radicals play an important role on the formation of DCNM from MA, DMA and PolyDADMAC. Among three types of amines, the potential of MA to form DCNM was the largest. According to the analysis of various nitrogen during UV/chlorine disinfection, it showed that amines were decomposed into the inorganic matter of NH_3_-N and NO_3_^−^-N. The study could be very useful to reduce the formation of HNMs through controlling disinfection conditions and to improve the disinfection methods for drinking water and sewage.

## Supplementary information

Supplementary information
