# Supplementary material for: Effects of amines on the formation and photodegradation of DCNM under UV/chlorine disinfection
Source: Sci Rep. 2020 Jul 28;10:12602. doi: 10.1038/s41598-020-69426-9 (PMC7387445; doi:10.1038/s41598-020-69426-9)
Supplement: Supplementary file 1 — Supplementary information [file 41598_2020_69426_MOESM1_ESM.docx]

**Supplementary Information for**

**Effects of amines on the formation and photodegradation of DCNM under UV/chlorine disinfection**

Lin Deng*, Xueying Liao, Jiaxin Shen, Bohui Xu

Department of Municipal Engineering, Southeast University, Nanjing, 211189, People Republic of China

** Corresponding author*

*Phone: (0086)13951974771*

*Email address:* [*dlwhu@163.com*](mailto:dlwhu@163.com)

**Text S1. Chemicals and reagents**

All chemicals were at least of analytical grade except as noted. Standard-grade of dichloronitromethane (DCNM) (97%) was from Quality Control Chemicals inc. (USA). Methylamine (MA) hydrochloride, dimethylamine (DMA) hydrochloride and Nessler's reagent were purchased from Aladdin Chemical Trading Co., Ltd. (Yixin, China). Poly-diallyl dimethyl ammonium chloride (PolyDADMAC) and sodium sulfamate (H_2_NNaSO_3_) were obtained from Yuanye Biotechnology Co., Ltd. (Shanghai, China). Sodium chloride (NaCl), methyl tert-butyl ether (MTBE), disodium hydrogen phosphate (Na_2_HPO_4_), sodium dihydrogenphosphate (NaH_2_PO_4_), tert-butyl alcohol (TBA) and hydrochloric acid (HCl) were obtained from Sinopharm Chemical Reagent Co., Ltd. (Shanghai, China). Sodium hypochlorite (NaOCl), sodium thiosulfate (Na_2_S_2_O_3_) were purchased form Kermel Chemical Reagent Co., Ltd. (Tianjin, China). Potassium persulfate (K_2_S_2_O_8_) from Sigma-Aldrich (St. Louis, MO, USA). Potassium sodium tartrate was purchased form Chron Chemical Reagent (Chengdu, China). All solutions were prepared using ultra-pure water produced by a Milli-Q water purification system (Billerica, MA).

**Text S2. Configuration and determination of DCNM standard sample**

To prepare a DCNM standard stock solution, a certain amount of DCNM standard substance was weighed and dissolved in 250 mL methyl tert-butyl ether (MTBE). A series of DCNM solutions (1000.0，500.0，400.0，200.0，100.0，50.0，20.0，10.0 μg/L) was prepared to analysed by Agilent 7890A GC equipped with ECD and HP-1 column (30 m × 320 mm × 0.25 μm). According to the results of the standard samples, the calibration curve equation for DCNM was Y_Area_=264.42C_DCNM_+412.88, R^2^=0.9994, and the detection limit of DCNM was 0.1 μg/L.

DCNM in water samples was extracted with 2 mL of methyl tert-butyl ether (MTBE), shook for 10 minutes, and then allowed to stand for 5 min until the mixture was layered. After that, 1 mL of the upper extracted solution was taking to a gas chromatography (GC) vial. Determination of DCNM in water samples according to USEPA 551.1 method ^1^, and DCNM recovery (98%) also showed good reliability and reproducibility of the method.

**Text S3. Determination methods for various forms of nitrogen**

Alkaline potassium persulfate digestion UV-spectrophotometry method was used for determination of TN according to the reference of HJ 636-2012^2^. NH_3_-N was determined by Nessler's reagent spectrophotometry according to the reference of HJ 535-2009^3^. NO_3_^-^-N and NO_2_^-^-N were determined by ultraviolet spectrophotometry according to the reference of HJ/T 346-2007^4^.

**Table S1. Kinetic fitted equation of DCNM degradation reaction under different UV intensity conditions**

| UV intensity | Fitted equation | *k*_obs,T_ (min^-1^) | R^2^ |
| --- | --- | --- | --- |
| Dark | -ln(C_t_/C_0_)=0.0045t+0.0017 | 0.0045 | 0.9898 |
| 5 W | -ln(C_t_/C_0_)=0.0833t+0.5450 | 0.0833 | 0.9847 |
| 10W | -ln(C_t_/C_0_)=0.1127t+0.0729 | 0.1127 | 0.9955 |
| 15W | -ln(C_t_/C_0_)=0.1297t+0.0853 | 0.1297 | 0.9948 |

**References:**

1. US Environmental Protection Agency. Determination of Chlorination Disinfection Byproducts and Chlorinated Solvents in Drinking Water by Liquid–Liquid Extraction and Gas Chromatography with Electroncapture Detection (Method 551.1). (1990).
2. China Environmental Protection Ministry. Water quality-Determination of total nitrogen-Alkaline potassium persulfate digestion UV spectrophotometric method (HJ 636-2012). (2012)
3. China Environmental Protection Ministry. Water quality-Determination of ammonia nitrogen-Nessler’s reagent spectrophotometry (HJ 535-2009). (2009).
4. China Environmental Protection Ministry. Water quality-Determination of nitrate-nitrogen-Ultraviolet spectrophotometry (HJ/T 346-2007). (2007).
